# Supplementary material for: Rapid, low-cost colorimetric detection of Salmonella Typhi bacteriophages for environmental surveillance
Source: mBio. 2025 Aug 18;16(9):e01963-25. doi: 10.1128/mbio.01963-25 (PMC12421811; doi:10.1128/mbio.01963-25)
Supplement: Fig. S1 — Effect of incubation temperature and incubation time on the performance of the colorimetric phage assay. [file mbio.01963-25-s0001.pdf]

<table>
<tr><td></td><td colspan="3">Conditions</td></tr>
<tr><td>Incubation Time</td><td>Control (37ºC)</td><td>Room Temperature (25ºC)</td><td>High Temperature (42ºC)</td></tr>
<tr><td>1.5 hrs</td><td>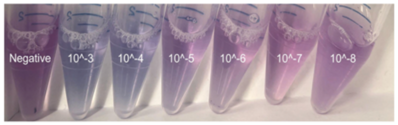
</td><td>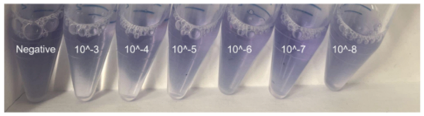
</td><td>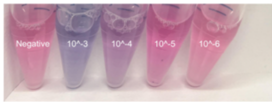
</td></tr>
<tr><td>2 hrs</td><td>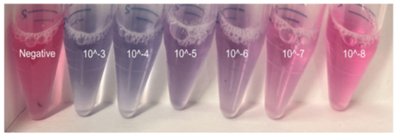
</td><td>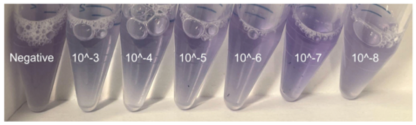
</td><td>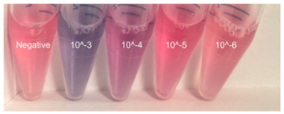
</td></tr>
<tr><td>2.5 hrs</td><td>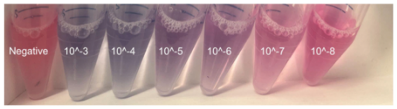
</td><td>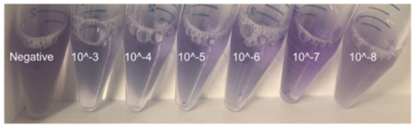
</td><td>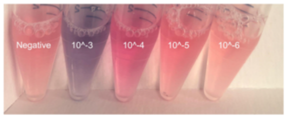
</td></tr>
<tr><td>3 hrs</td><td>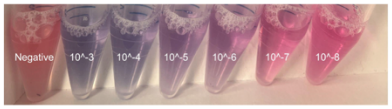
</td><td>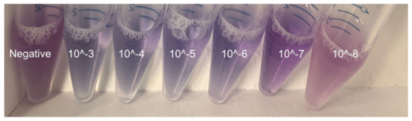
</td><td>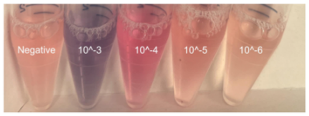
</td></tr>
<tr><td>3.5 hrs</td><td></td><td>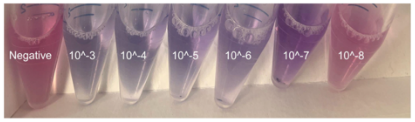
</td><td></td></tr>
<tr><td>5 hrs</td><td>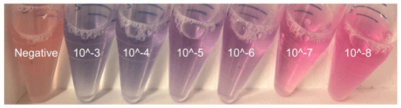
</td><td>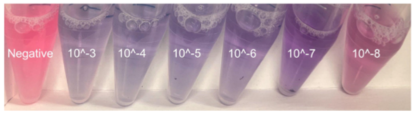
</td><td>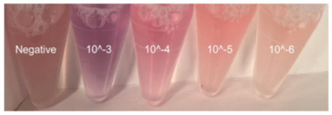
</td></tr>
</table>

Figure S1. Effect of incubation temperature and incubation time on the performance of the colorimetric phage assay. Color development in the colorimetric assay was monitored at different temperatures (25°C and 37°C and 42°C) over a range of incubation times. Several phage concentrations (2.9 to $2.9x10^7$ PFU/ml) were tested and cultures contained LB media, *S*. Typhi Ty2 strain, Vi phage dilution and resazurin. Negative control, contains LB media, *S*. Typhi Ty2 strain and resazurin.
